# Janus *N*,*N*-dimethylformamide as a solvent for a gradient porous wound dressing of poly(vinylidene fluoride) and as a reducer for *in situ* nano-silver production: anti-permeation, antibacterial and antifouling activities against multi-drug-resistant bacteria both *in vitro* and *in vivo*†

**DOI:** 10.1039/c8ra03234c

**Published:** 2018-07-25

**Authors:** Menglong Liu, Ying Wang, Xiaodong Hu, Weifeng He, Yali Gong, Xiaohong Hu, Meixi Liu, Gaoxing Luo, Malcolm Xing, Jun Wu

**Affiliations:** Institute of Burn Research, State Key Laboratory of Trauma, Burn and Combined Injury, Southwest Hospital, Third Military Medical University (Army Medical University) Chongqing 400038 China logxw@yahoo.com malcolm.xing@umanitoba.ca editorinchief@burninchina.com +86-23-65461677 +86-23-68754173; State Key Laboratory of Polymer Materials Engineering, Polymer Research Institute of Sichuan University Chengdu 610065 China; Department of Burns, The First Affiliated Hospital, SunYat-Sen University Guangzhou 510080 China; Department of Mechanical Engineering, University of Manitoba Winnipeg MB R3T 2N2 Canada

## Abstract

The requirements for anti-permeation, anti-infection and antifouling when treating a malicious wound bed raise new challenges for wound dressing. The present study used *N*,*N*-dimethylformamide to treat poly(vinylidene fluoride) (PVDF) in order to obtain a dressing impregnated with *in situ* generated nano-silver particles (NS) *via* an immersion phase inversion method. Scanning electron microscopy (SEM) images showed that the film was characterized by a two-layer asymmetric structure with different pore sizes (top layer: ∼0.4 μm; bottom layer: ∼1.8 μm). The moisture permeability test indicated that the film had an optimal water vapor transmission rate (WVTR: ∼2500 g m^−2^ per day). TEM images revealed the successful formation of spherical NS, and Fourier-transform infrared spectroscopy (FTIR) demonstrated the integration of PVDF and NS (*i.e.*, PVDF/NS). Correspondingly, the water contact angle measurements confirmed increased membrane surface hydrophobicity after NS integration. The inductively coupled plasma (ICP) spectrometry showed that the PVDF/NS displayed a continuous and safe release of silver ions. Moreover, *in vitro* experiments indicated that PVDF/NS films possessed satisfactory anti-permeation, antibacterial and antifouling activities against *A. baumannii* and *E. coli* bacteria, while they exhibited no obvious cytotoxicity toward mammalian HaCaT cells. Finally, the *in vivo* results showed that the nanoporous top layer of film could serve as a physical barrier to prevent bacterial penetration, whereas the microporous bottom layer could efficiently prevent bacterial infection caused by biofouling, leading to fast re-epithelialization *via* the enhancement of keratinocyte proliferation. Collectively, the results show that the PVDF/NS25 film has a promising application in wound treatment, especially for wounds infected by multi-drug-resistant bacteria such as *A. baumannii*.

## Abstract 2

The requirements for anti-permeation, anti-infection and antifouling when treating a malicious wound bed raise new challenges for wound dressing.

## Graphical abstract

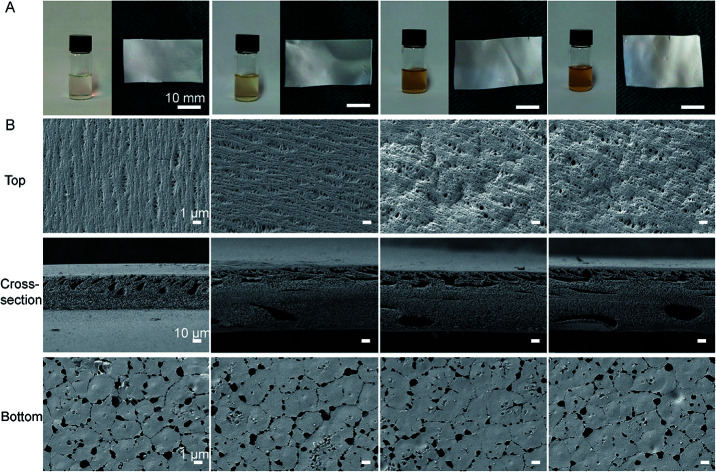

## 1. Introduction

Skin plays a critical role in protecting the internal organs.^1^ When damaged, serious body fluid loss and microorganism invasion can occur, which may lead to acid–base disturbance and severe infection.^2,3^

Wound dressing is an important treatment option for cutaneous wounds as it can temporarily reconstruct a mechanical barrier and help to regenerate dermal and epidermal tissues.^4,5^ A dressing should generally be composed of a porous structure and have a suitable water vapor transmission rate (WVTR), to avoid wound maceration and festering but to maintain a moist microenvironment to promote re-epithelialization.^5–7^ However, oversized pores in dressings could become a pathway for the invasion of external bacteria during the wound healing process, which would lead to the loss of the barrier function. Previous studies have indicated that a traditional gauze containing large pores is easily permeable to exogenous bacteria, and associated with a high infection rate.^8,9^ Thus, in order to guarantee the efficient barrier function against bacteria, a small pore size in the outer layer of a wound dressing is necessary. Considering the size of bacteria (0.5–5 μm), the optimal outer pore size should be below 0.5 μm.^10,11^ In addition, our previous work has suggested that pore sizes ranging from 1.2 to 2.1 μm are beneficial for controlling water loss and keeping a moist microenvironment to accelerate re-epithelialization.^12,13^ Nevertheless, a wound dressing characterized by a gradient porous structure to effectively prevent bacterial permeation while maintaining a moist wound bed has not been reported yet.

As well as optimizing the porous structure, preventing microbial contamination is a great challenge for wound dressings.^14^ If a wound dressing is contaminated by pathogenic bacteria and then used to cover a wound, it causes serious infection and delays the healing process. This scenario not only leads to the failure of medical treatment, but also probably contributes to the emergence of drug resistance.^15,16^ Therefore, it is very important to endow the dressing with intrinsic excellent antibacterial and antifouling activities. A promising strategy employed to prevent bacterial fouling is the addition of antibacterial nanoparticles to the membrane backbone materials during film fabrication.^17–19^ Among these, nano-silver particles (NS) could be a promising candidate. NS (1–100 nm) has broad-spectrum antimicrobial activity and outstanding killing efficacy towards microorganisms including multi-drug-resistant bacteria (MDR).^20,21^ Specifically, NS kills bacteria through various mechanisms, including the disruption of the bacterial membrane, interference with bacterial DNA replication and inhibition of respiratory activity, which efficiently destroy bacteria without inducing resistance.^22^

As a synthetic fluoropolymer, poly(vinylidene fluoride) (PVDF) has remarkable chemical stability and thermal performance as well as good biocompatibility.^23^ Pristine PVDF and modified PVDF films have been widely applied in wastewater treatment, drug delivery, electronic devices and sensor preparation.^24–27^ In recent years, Lins *et al.* found that PVDF electrospun fiber could accelerate the growth and differentiation of neural stem cells.^28^ He *et al.* reported that an electrospun PVDF membrane integrated with an enrofloxacin composite showed excellent antibacterial activity and a favorable healing effect on skin wounds.^29^ These studies suggest the feasibility of using PVDF as a potential wound dressing. However, the fabrication of electrospun fibers needs specialized equipment, and the electrospinning technology is relatively complicated. Actually, PVDF is a semi-crystalline polymer which possesses good film forming ability, and a porous PVDF film can be easily prepared using the immersion phase inversion method.^30,31^ Furthermore, the structure of the formed film can be improved by adjusting the polymeric concentration.^32^ Nevertheless, applying a PVDF film prepared by the phase inversion method as a wound dressing is still rare.

Hence, in this work, we aimed to prepare a composite film of PVDF integrated with NS that consisted of a double layer with a gradient porous structure using a facile immersion phase inversion method. Janus *N*,*N*-dimethylformamide (DMF) was selected as the solvent during the fabrication process, as it could also act to reduce Ag^+^ to NS in mild conditions,^33^ so that the PVDF/NS film could be prepared *in situ* by a one-step phase inversion method. The phrase “Janus DMF” used here means that the DMF plays the dual roles of solvent and reducing agent. We hypothesized that the nanoporous top layer of PVDF/NS film would serve as a physical barrier to prevent bacterial permeation during the wound healing process, while the microporous bottom layer of the PVDF/NS film which contacted the wound could prevent bacterial infections induced by surface biofouling.

## 2. Materials and methods

### 2.1 Materials and animals

Poly(vinylidene fluoride) powder (PVDF, Mw = 70 000) and *N*,*N*-dimethylformamide (DMF) were purchased from Sigma-Aldrich (MO, USA). Silver nitrate (AgNO_3_, 99%) was purchased from Sangon (Shanghai, China). The multi-drug-resistant *Acinetobacter baumannii* (*A. baumannii*, ATCC 19606) and non-drug-resistant *Escherichia coli* (*E. coli*, ATCC 25922) bacterial strains were provided by the Microbiology Laboratory, Institute of Burn Research, Southwest Hospital, Third Military Medical University (TMMU, Chongqing, China).

BALB/c adult mice (male, 20–25 g, 8–10 weeks old, specific pathogen free (SPF) level) were supplied by the Experimental Animal Department of the Third Military Medical University (TMMU). All animal protocols were permitted by the Institutional Animal Care and Use Committee of TMMU (Chongqing, China), and all animal experiments were performed in strict accordance with the Regulation on the Management of Laboratory Animals, which was approved by the Chinese Association for Laboratory Animal Sciences (CALAS). The animals were individually housed in plastic cages under standardized conditions (circadian rhythm: 12 h; room temperature: 25 °C; relative humidity: 50%).

### 2.2 Preparation of the PVDF and PVDF/NS films

The pristine PVDF and PVDF/NS films were synthesized using the immersion phase inversion method as previously described.^34^ The preparation of a film by the immersion phase inversion method is a process of liquid–liquid separation. During the process, the film-forming solution is divided into a polymer-rich phase and a polymer-poor phase, which finally constitute the structural scaffold and the pores of the solid film, respectively. As the phase inversion rates of the top surface and bottom surface are different when the cast solution is immersed in coagulation liquid, the generated pore sizes of the two sides are also different, which leads to the formation of an asymmetrical porous film.^35–37^ The increase of polymeric concentration can enhance the viscosity of the cast solution, and influence the phase inversion rate.^32,38^ Therefore, the porous structure of the formed film can be controlled by adjusting the polymeric concentration. To prepare different concentrations of PVDF films, 1 g (5 wt%), 2 g (10 wt%) or 4 g (20 wt%) of PVDF powder were added into 20 mL DMF solution. Each solution was vigorously stirred for 30 min at 60 °C, and then reacted in a 37 °C shaker incubator for 24 h. Subsequently, the mixture solutions were placed in a vacuum oven for 1 h at 50 °C to remove air bubbles. The harvested solutions were uniformly cast onto Petri dishes and immediately immersed into a DDH_2_O coagulating bath at room temperature. After 30 min, the cast films were successively rinsed with 75% alcohol and DDH_2_O to eliminate the solvent.^25^ Finally, the films were dried in a vacuum oven at 40 °C overnight and sterilized by ultraviolet light radiation. To prepare PVDF/NS films, different final concentrations of AgNO_3_ (*i.e.*, 10 mM, 25 mM, 50 mM) were each firstly dispersed in 20 mL DMF. After 2 g (10 wt%) PVDF powder was added into each solution, the PVDF/NS films were synthesized as described above using the immersion phase inversion method. The PVDF integrated with different concentrations of NS were designated as PVDF/NS10, PVDF/NS25 and PVDF/NS50.

### 2.3 Characterization of the PVDF/NS films

The morphologies of the PVDF/NS films were observed using scanning electron microscopy (SEM, Crossbeam 340, Zeiss, Germany), and the average pore size (*n* = 50 pores) and thickness of each film were measured using Image J software. The morphology of the synthetic NS was observed using transmission electron microscopy (TEM, Zeiss LIBRA 200 FEG, Germany) by dropping 5 μL of mixture solution onto a carbon-coated copper grid. The Fourier transform infrared (FTIR) spectra of PVDF, PVDF/NS10, PVDF/NS25 and PVDF/NS50 films were acquired using a PerkinElmer FTIR spectrometer (100S). The water contact angles were detected using a contact angle analyzer (Theta Lite 101, Biolin Scientific, Sweden).

### 2.4 Determination of water vapor transmission rate (WVTR)

To detect the moisture permeability of the PVDF/NS films, the WVTR was measured on the basis of the American Society for Testing and Materials (ASTM) standard.^12^ In brief, a specimen was cut into a disc (diameter: 35 mm) and placed on the mouth of a cylindrical cup containing DDH_2_O. Then, the specimen and cup were sealed by Teflon tape and positioned in an incubator which was set to 37 °C and 90% relative humidity. The data was automatically recorded and analyzed by the WVTR tester (W3/030, Labthink, China). All the measurements were repeated three times.

### 2.5 Bacterial permeation test

The commercial bacteria used in this test were *A. baumannii* and *E. coli*. Briefly, a bacterial colony of *A. baumannii* or *E. coli* was firstly inoculated overnight in 4 mL of Luria-Bertani (LB) medium with shaking at 37 °C. Then the log-phase bacterial solution was diluted in LB medium to acquire a concentration of 1 × 10^8^ colony forming units (CFU) mL^−1^ for the following experiments.^12^ The sterilized films (10 × 10 mm) of Vaseline gauze, PVDF, PVDF/NS10, PVDF/NS25 and PVDF/NS50 were each washed with phosphate-buffered saline (PBS; pH 7.4), and then placed on the surface of an agar plate. Subsequently, 25 μL of diluted bacterial suspension was dropped onto the center of each film, and incubated at 37 °C for 24 h. Then the agar under each film was carefully cut out with a knife and immersed into 5 mL of PBS. After sonication for 5 min, the agar was removed and the solution was serially diluted with PBS to a 2000× dilution. Finally, 20 μL of diluted solution was uniformly coated on an agar plate and incubated at 37 °C for 18 h to count the number of bacteria.

### 2.6 Bacterial suspension assay

The bacterial suspension assay was performed as previously described.^39^ Briefly, the log-phase bacterial solution was diluted in LB medium to acquire the required starting concentration (optical density at 600 nm; OD_600_ = 0.07). Subsequently, 500 μL of diluted bacterial suspension was dispensed into each well of a 24-well plate, and a sterilized sample of film (10 × 10 mm) was placed into the bacterial suspension. Then the 24-well plate was incubated in a 37 °C shaker at 50 rpm. After 24 h, 100 μL of bacterial suspension from each well was added into a 96-well plate and the OD_600_ value was measured. In addition, the bacterial suspension was further diluted 20 000× with PBS solution, and 20 μL of the diluted bacterial suspension was uniformly plated onto agar, and incubated at 37 °C for 18 h. Then the agar plate was photographed and the number of bacterial colonies was counted. Here, a bacterial suspension without any treatment (labeled as control) and pristine PVDF were used as negative control groups. The antibiotics streptomycin, tetracycline and ceftazidime, belonging to the aminoglycoside, tetracycline and β-lactam antibiotics, respectively, have been widely used in clinical practice, so they were selected as positive control groups. The detailed working concentrations of streptomycin (1000 μg mL^−1^), tetracycline (20 μg mL^−1^) and ceftazidime (50 μg mL^−1^) were adopted from previous literature reports.^39–41^

### 2.7 Antifouling test

The antifouling effects of PVDF/NS films were investigated using *A. baumannii*. Firstly, 200 μL of bacterial suspension standardized to 0.5 McFarland standards (10^8^ CFU mL^−1^) was added into each well of a 24-well plate. Secondly, sterilized PVDF, PVDF/NS10, PVDF/NS25 or PVDF/NS50 film (8 × 8 mm) was placed onto the bacterial suspension, such that the bottom surface of the sample was in contact with the liquid. Thirdly, after incubation at 37 °C for 1 h, the sample was collected and placed into 1 mL of PBS solution, and sonicated for 5 min to detach the adherent bacteria. Finally, the PBS solution was diluted 200× and 20 μL of the diluted solution was plated onto agar. After 18 h, the agar plate was photographed and the number of bacterial colonies was counted.

### 2.8 *In vitro* cytotoxicity evaluation

The leach liquors of PVDF, PVDF/NS10, PVDF/NS25 and PVDF/NS50 films were extracted according to ISO 10993-5. Briefly, a sterilized sample (1.4 cm diameter) was immersed in 1 mL of RPMI1640 medium (Gibco, USA) containing 10% fetal bovine serum (FBS, Gibco, USA) at 37 °C for 24 h. Then the medium was filtered with a sterilized filter (pore size: 0.22 μm) to remove microorganisms, and reserved for later use. To observe the cell morphology after treatment with PVDF/NS, 10 000 HaCaT cells were firstly inoculated into a 24-well plate with prepared glass coverslips and placed in a 37 °C incubator for 24 h. Then the medium was replaced with 500 μL of either leach liquor, RPMI1640 medium containing 10% FBS or RPMI1640 medium containing 10% FBS and 0.5% (v/v) Triton-X 100. The cell inoculated with RPMI1640 medium containing 10% FBS served as the negative control group, while that treated by RPMI1640 medium containing 10% FBS and 0.5% (v/v) Triton-X 100 served as the positive control group. At 1, 2 and 3 days post-seeding, the cells were rinsed with PBS, then fixed in 4% paraformaldehyde at 25 °C for 30 min, stained with phalloidin (100 ng mL^−1^) for 1 h at 37 °C and DAPI for 30 s at 25 °C. Finally, the cells were observed under a fluorescence microscope (Olympus, Japan). To further detect the cell viability of the HaCaT cells after treatment with PVDF/NS, the CCK8 test was used. Briefly, 3000 cells were seeded into each well of a 96-well plate and incubated for 24 h. Then the medium was replaced with 100 μL of either leach liquor, RPMI1640 medium containing 10% FBS or RPMI1640 medium containing 10% FBS and 0.5% (v/v) Triton-X 100. At 1, 2 and 3 days post-seeding, the liquid was removed and 100 μL of RPMI1640 medium containing 10 μL CCK8 solution was added to each well. After incubation at 37 °C for 2 h, the optical density at 450 nm was measured by an enzyme-linked immunosorbent assay reader (Thermo Varioskan Flash, USA). The cell viability was calculated as follows:Cell viability% = (OD^*n*^ − OD^−^)/(OD^+^ − OD^−^) × 100%where OD^*n*^ represents the OD value of PVDF, PVDF/NS10, PVDF/NS25, PVDF/NS50 or the positive control groups, OD^+^ represents the OD value of the negative control group, and OD^−^ represents the OD value of RPMI1640 medium containing 10% FBS without cells.

### 2.9 Release of silver (Ag^+^) ions

To detect the release of Ag^+^ from the sample, a piece of either PVDF/NS10, PVDF/NS25 or PVDF/NS50 film (10 × 10 mm) was placed into 6 mL of PBS (pH 7.4 or pH 6.3) at 37 °C. Then the supernatant was harvested at each determined time, *i.e.* days 1, 2, 3, 4, 5, 6, 7 and 8, and analyzed using inductively coupled plasma-atomic emission spectrometry (ICP-AES, Leeman, USA).

### 2.10 *In vivo* animal study

The *in vivo* study was performed using an infectious murine full-thickness skin defect wound model based on previous studies with slight modification.^42,43^ There were four animal groups in the test, namely the Blank group, Vaseline gauze group, PVDF group and PVDF/NS25 group, and five mice were used in each group. In brief, the sterilized samples (8 × 8 mm) of Vaseline gauze, PVDF and PVDF/NS25 were each pre-seeded with a 25 μL bacterial suspension of *A. baumannii* which was standardized to 0.5 McFarland standards (10^8^ CFU mL^−1^), then air-dried for 10 min before use. Each BALB/c mouse was anesthetized with an intraperitoneal injection of 1% pentobarbital, the dorsal surface was shaved and cleaned with 75% alcohol, and then two 6 mm-diameter full-thickness wounds were created on the back and photographed to represent the starting wound area. Subsequently, the bacteria-seeded side of the prepared Vaseline gauze, PVDF or PVDF/NS25 film was used to cover the wounds and then fixed with an adhesive biological membrane. Mice that were not exposed to bacteria were set as the Blank group. At days 3, 5 and 7 post-surgery, the wounds were photographed and the films were changed. The reserved wound areas were carefully measured using IPP 6.0 software. The percentage of closed wound area was calculated using the following formula:% of closed wound area = (*I* − *R*)/*I* × 100%where *I* indicates the number of pixels of the starting wound area, and *R* indicates the number of pixels of the reserved wound area at the predetermined time. At day 7 post-surgery, the wounds of the mice in each group were harvested and homogenized in 5 mL of PBS (pH 7.4), and then the CFU of bacteria were counted by spreading the diluted PBS solution onto agar plates, as described for the *in vitro* antibacterial test.

Additionally, another four mice from each group were used for histological examination. At days 3 and day 5 post-surgery, the cutaneous wound tissues were harvested and fixed with 4% paraformaldehyde for 24 h. Then the tissues were embedded in paraffin and stained with hematoxylin and eosin (H&E). The length of the newly formed epidermis, which is defined as the distance from the border between the undamaged skin tissue and the wound area to the leading edges of the newly formed epidermis,^44^ and the number of inflammatory cells infiltrated into the subcutaneous area of the wound edge were measured using Image J software. All measurements using software were performed by two independent pathologists.

To evaluate the *in vivo* toxicity, the wounds of mice (five mice in each group) were either exposed (no cover at all), or covered by Vaseline gauze, PVDF or PVDF/NS25 films for 7 days. Then, the mice were sacrificed and the major organs including the heart, liver, spleen, lung and kidney were harvested for histological analysis as previously described.^45,46^

### 2.11 Immunohistochemistry

The immunohistochemistry assay was performed as previously described.^12^ Briefly, after deparaffinized, rehydrated, and heat-mediated antigen retrieval, the sections were blocked using 10% normal goat serum (Zhongshan Biology Company, China) at 37 °C for 30 min, and then incubated with anti-PCNA antibody (ab15497, 1 : 200 dilution, Abcam, UK) at 4 °C overnight. After being washed with PBS, the sections were successively incubated with biotinylated goat-anti-rabbit IgG antibody (Zhongshan Biology Company, China) and avidin peroxidase reagent (Zhongshan Biology Company, China) at 37 °C for 30 min. Then the sections were stained with 3,3′-diaminobenzidine tetrahydrochloride and hematoxylin. Finally, images of sections were acquired using an optical microscope (CTR6000, Leica, Germany), and the counting of PCNA-positive keratinocytes per field in the newly generated epidermis was performed by two independent pathologists.

### 2.12 Statistics

All the data are expressed as mean ± standard deviation (SD) and statistically compared using the one-way ANOVA. *P* < 0.05 was accepted as statistically significant (*represents *P* < 0.05 and ** represents *P* < 0.01). The statistical software used in this paper was SPSS 13.0 (SPSS Inc., Chicago, IL, USA).

## 3. Results and discussion

### 3.1 Optimization of polymeric concentration

As the polymeric concentration has the predominant influence on the structure of the formed membrane when using the phase inversion method,^47,48^ SEM analysis was firstly employed to optimize this. Fig. S1 (ESI†) displays the SEM images of the pristine PVDF films prepared with different polymeric concentrations. The 5 wt% PVDF film exhibited a heterogeneous structure consisting of a disordered top surface and a rough bottom surface, which implied that such a low concentration of polymer was insufficient for membrane formation by phase inversion.^32,47^ However, when the polymeric concentration was increased up to 20 wt%, the pore formation was obviously limited in the top layer of the PVDF film because of the viscosity enhancement during the synthetic process.^49^ In contrast, both the top layer and the bottom layer of 10 wt% PVDF film showed porous structures with relatively uniform pore sizes. As we all know, an ideal wound dressing should have high porosity for moisture and oxygen exchange, since there is no doubt that a porous film benefits wound healing.^6,7^ Thus, based on the SEM results, the 10 wt% polymeric concentration was selected as the optimal one for integration with NS because this film was characterized by homogeneity, smoothness and a porous structure.

### 3.2 Formation and structures of PVDF/NS films

**Fig. 1:**
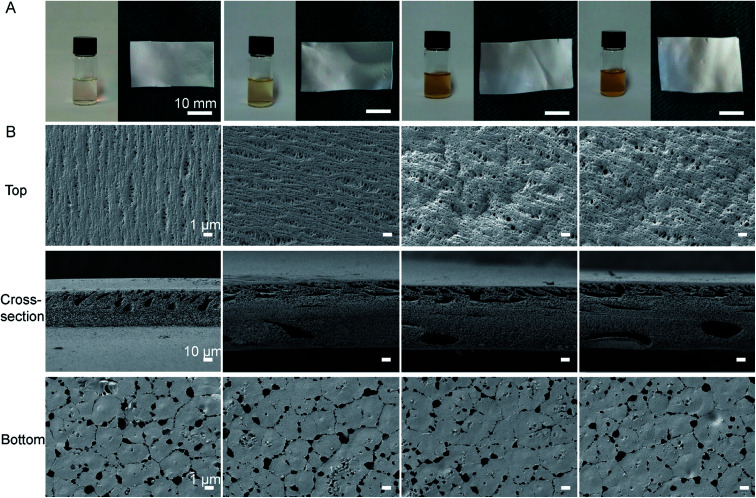
(A) Macroscopic appearances of solutions and the corresponding formed films of PVDF, PVDF/NS10, PVDF/NS25 and PVDF/NS50 (from left to right) after 24 h incubation. (B) SEM images of PVDF, PVDF/NS10, PVDF/NS25 and PVDF/NS50 films. Magnification of top surface and bottom surface images: ×3000; magnification of cross-section images: ×300.

As shown in Fig. 1A, the color of the polymeric solution changed from pale white to yellow after AgNO_3_ was added into the DMF solution, which suggested that the Ag^+^ was reduced to the zerovalent metal.^25^ TEM results further showed that the synthetic NS particles had typical spherical morphology and the diameter of the NS particles was well below 10 nm (Fig. S2, ESI†). These results confirmed that DMF was not only a favorable solvent, but also an active reducing agent for NS production in the formation of PVDF/NS, as previously described.^33,50^

Fig. 1B revealed the surface and cross-sectional morphologies of PVDF and PVDF/NS films. Similar to the morphology of pristine PVDF, the PVDF/NS10, PVDF/NS25 and PVDF/NS50 films were characterized by a typical asymmetric structure composed of a nanoporous top layer and a microporous bottom layer. The pore sizes of the top surface and bottom surface and the thickness of each film are listed in Table 1. As expected, the average pore sizes of the top surfaces were well below 0.4 μm, suggesting that this layer is enough to serve as a physical barrier to prevent bacterial penetration (size of bacteria: 0.5 μm–5 μm).^10,11^ Meanwhile, the bottom layer containing pores with a size of 1.8 μm might contribute to controlled water loss.^12,13^ The cross-sectional SEM images showed a spongy structure of pristine PVDF and modified PVDF/NS films, as Xiao *et al.* have previously described.^51^ Such an interconnected fibrous structure in the microporous matrix could provide enough interspace for gas and water exchange. The result of WVTR measurements further confirmed this. Table 1 reveals that the average WVTRs of PVDF, PVDF/NS10, PVDF/NS25 and PVDF/NS50 films were 2507.9 g m^−2^ per day, 2500.5 g m^−2^ per day, 2490.5 g m^−2^ per day and 2486.3 g m^−2^ per day, respectively. These data are close to the optimal WVTR (2028.3 g m^−2^ per day) reported before, which indicated that the as-prepared samples would be beneficial for maintaining a moist environment to promote re-epithelialization when applied to wound management.^52^ The thicknesses of the PVDF/NS films were larger than that of pristine PVDF film, which might be due to the deposition of NS.^25^ In addition, the SEM images showed that the NS nanoparticles were deposited on the bottom surfaces of PVDF/NS10, PVDF/NS25 and PVDF/NS50 films, which further confirmed the formation and integration of NS.

**Table 1:** The pore size, WVTR and thickness of pristine PVDF, PVDF/NS10, PVDF/NS25 and PVDF/NS50 films

	Pore size (μm) of top layer	Pore size (μm) of bottom layer	WVTR (g m^−2^ per day)	Thickness (μm)
PVDF	0.32 ± 0.07	1.93 ± 0.45	2507.9 ± 200.2	113.8 ± 8.6
PVDF/NS10	0.33 ± 0.09	1.88 ± 0.37	2500.5 ± 193.8	148.5 ± 5.8
PVDF/NS25	0.35 ± 0.09	1.88 ± 0.36	2490.5 ± 190.0	142.7 ± 3.9
PVDF/NS50	0.35 ± 0.10	1.87 ± 0.48	2486.3 ± 192.3	149.9 ± 3.2

**Fig. 2:**
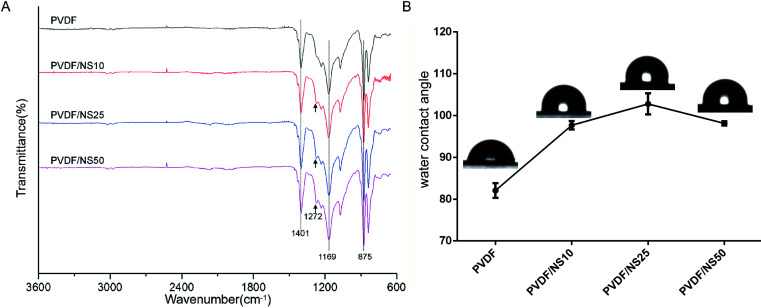
(A) FTIR spectra and (B) water contact angles of PVDF, PVDF/NS10, PVDF/NS25 and PVDF/NS50 films. The black arrows indicate the new absorption peak in samples after NS integration.

To further explore the chemical structures of PVDF and PVDF/NS films, FTIR analysis was performed. As shown in Fig. 2A, two characteristic absorption peaks at 875 cm^−1^ and 1401 cm^−1^ in the PVDF spectrum indicated the stretching vibration of C–H bonds, while the peak at 1169 cm^−1^ corresponded to the stretching vibration of C–F bonds.^53–55^ After NS deposition, a new peak at 1272 cm^−1^ was observed in the spectra of PVDF/NS10, PVDF/NS25 and PVDF/NS50, indicating the interaction between NS and PVDF.^56^ Taken together, the macroscopic appearance, SEM, TEM and FTIR results demonstrated the successful fabrication of PVDF/NS films, composed of double layers with different pore sizes.

The surface wettability of the bottom surface of pristine PVDF and functionalized PVDF/NS films were assessed using water contact angle measurements. As shown in Fig. 2B, the water contact angle of pristine PVDF was 82.1° ± 1.8°, which indicated the intrinsic hydrophobic character of PVDF. After integration with NS, the water contact angles of PVDF/NS10, PVDF/NS25 and PVDF/NS50 films increased to 97.7° ± 1.0°, 102.8° ± 2.5° and 98.1° ± 0.5°, respectively, which might be due to the increased surface roughness as reported before.^57^

### 3.3 Ag^+^ release into PBS

**Fig. 3:**
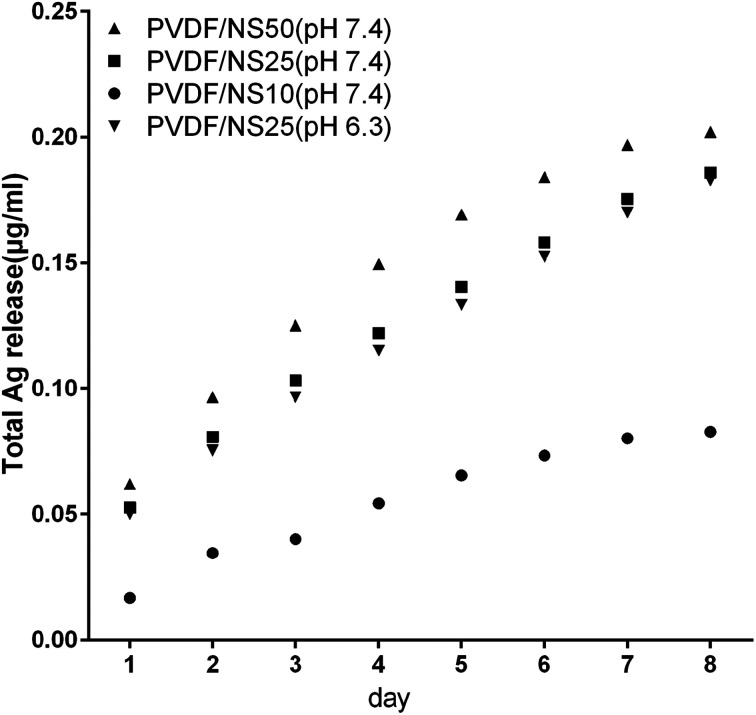
Silver ion release in PBS (pH 7.4) from PVDF/NS10, PVDF/NS25 and PVDF/NS50 films, and in PBS (pH 6.3) from the PVDF/NS25 film at days 1, 2, 3, 4, 5, 6, 7 and 8.

The antibacterial activity of NS-containing biomaterials is mainly attributed to the localized release of Ag^+^ from the core of the NS,^58,59^ so the Ag^+^ release profiles of the PVDF/NS composite films were detected using ICP-AES. As shown in Fig. 3, the amount of released Ag^+^ increased as the amount of NS in the film increased. A relatively fast release of Ag^+^ occurred at the beginning, and then the release rate gradually decreased with incubation time. In detail, the concentrations of released Ag^+^ were about 0.02 μg mL^−1^, 0.05 μg mL^−1^ and 0.06 μg mL^−1^ on day 1 for PVDF/NS10, PVDF/NS25 and PVDF/NS50 films, respectively. This data suggested that PVDF/NS25 and PVDF/NS50 films might have antibacterial activity because the bactericidal level of Ag^+^ is about 0.05 μg mL^−1^ in PBS.^60,61^ Furthermore, the continuous release of Ag^+^ was still observed after 8 days, which indicated that the PVDF/NS25 and PVDF/NS50 films possessed long-term antibacterial properties. More significantly, the total released Ag^+^ concentrations for the three PVDF/NS composite films after 8 days were all below 1 μg mL^−1^, which is far lower than the toxic concentration to the human body (10 μg mL^−1^),^62^ implying that such functional PVDF/NS films would have good biocompatibility. In addition, as bacterial infection sites are usually in an acidic environment,^63,64^ the concentration of released Ag^+^ from PVDF/NS25 into acidic solution (PBS, pH 6.3) was detected. The Ag^+^ release profile of PVDF/NS25 at pH 6.3 was approximately similar to that at pH 7.4, and the concentration of released Ag^+^ was still above the bactericidal concentration (0.05 μg mL^−1^) on day 1, indicating that PVDF/NS25 had antibacterial activity in an acidic environment. The amount of released Ag^+^ at pH 6.3 was slightly lower than that at pH 7.4, which might be because hydrogen ions (H^+^) inhibited Ag^+^ release to some extent.^65^ All together, the results revealed that PVDF/NS25 and PVDF/NS50 films were probably able to prevent bacterial infection over an extended time without causing significant toxicity to humans.

### 3.4 Anti-permeation activity of PVDF/NS films

**Fig. 4:**
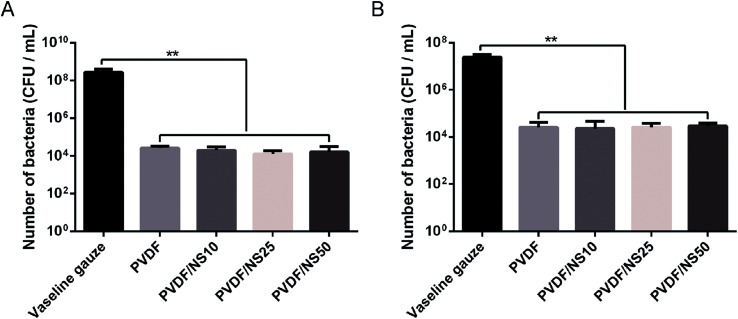
Bacterial penetration of Vaseline gauze, PVDF, PVDF/NS10, PVDF/NS25 and PVDF/NS50 films for (A) *A. baumannii* and (B) *E. coli*. (E) The values are shown as the mean ± SD (*n* = 3).

The small pore size of the top surface of the PVDF and PVDF/NS films implied that this layer of the films could serve as a physical barrier against bacterial invasion. Therefore, the anti-permeation activity of the PVDF/NS films was investigated using a bacterial permeation test. Obviously, pristine PVDF and functional PVDF/NS films showed much lower bacterial penetration than did the Vaseline gauze, both for *A. baumannii* and *E. coli* (Fig. 4), confirming that the top layer of the PVDF and PVDF/NS films efficiently prevented bacterial invasion from the external environment. This result suggested that the PVDF/NS films could effectively protect the wound from external bacterial invasion during the wound healing process. There were no significant differences in bacterial number among the PVDF and PVDF/NS groups; this might be because any bacteria that rapidly passed through the PVDF/NS films did not suffer from sufficient damage by NS as the toxicity of NS is time-dependent.^66,67^

### 3.5 *In vitro* antibacterial activity of PVDF/NS films

**Fig. 5:**
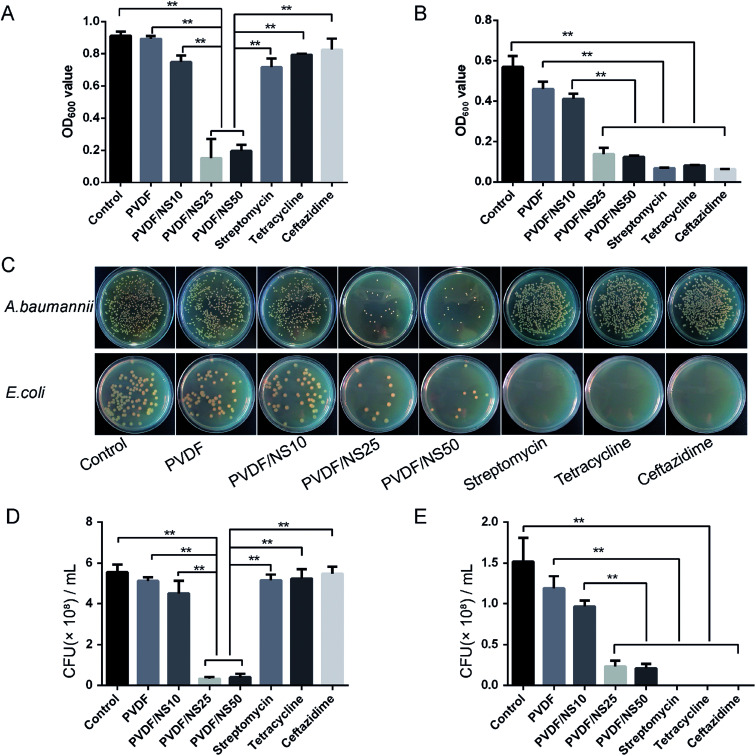
OD_600_ values of (A) *A. baumannii* and (B) *E. coli* bacterial suspensions after 24 h incubation with PVDF, PVDF/NS10, PVDF/NS25 or PVDF/NS50 films, or antibiotics. (C) Representative photographs of bacterial colonies of *A. baumannii* and *E. coli*. The quantitative number of bacteria for (D) *A. baumannii* and (E) *E. coli*. The values are shown as the mean ± SD (*n* = 3).

The antibacterial activity of the functional PVDF/NS films against both multi-drug-resistant bacteria (*A. baumannii*) and non-drug-resistant bacteria (*E. coli*) was detected by conducting a bacterial suspension assay. *A. baumannii* was selected as the multi-drug-resistant bacteria model here because it is one of the most common bacteria that causes hospital-acquired infections, and it is often resistant to a large number of antibiotics according to previous studies.^68,69^ As shown in Fig. 5A and B, the OD_600_ values of bacteria incubated with the PVDF/NS25 or PVDF/NS50 groups were significantly lower than those of bacteria incubated with the control, PVDF or PVDF/NS10 groups both for *A. baumannii* and *E. coli*, suggesting that PVDF/NS25 and PVDF/NS50 films could effectively inhibit the bacterial growth. The numbers of bacterial colonies counted from the co-cultured suspension were also significantly lower in the PVDF/NS25 and PVDF/NS50 groups than those in the control, PVDF and PVDF/NS10 groups (Fig. 5C–E), which further confirmed the excellent antibacterial activity of the PVDF/NS25 and PVDF/NS50 films. More significantly, both the OD_600_ values and numbers of bacterial colonies revealed that the PVDF/NS25 and PVDF/NS50 films had much higher antibacterial efficacies than three traditional antibiotics toward *A. baumannii*. This result suggested that the traditional antibiotics would be overwhelmed with *A. baumannii* infection, while the PVDF/NS25 and PVDF/NS50 films could effectively eliminate it. Moreover, NS-containing biomaterials fight against bacteria *via* several different mechanisms (*i.e.* cell membrane disruption, DNA damage and induction of reactive oxygen species release),^22^ so potential drug-resistance issues could well be avoided. Collectively, based on these results, PVDF/NS25 and PVDF/NS50 films were proven to be valuable new candidates for the treatment of bacterial infections, especially for multi-drug-resistant bacteria such as *A. baumannii*.

### 3.6 *In vitro* antifouling activity of PVDF/NS films

**Fig. 6:**
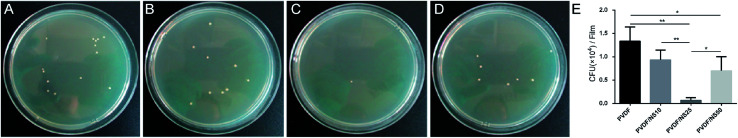
Antifouling activities of (A) PVDF, (B) PVDF/NS10, (C) PVDF/NS25 and (D) PVDF/NS50 films. The values are shown as the mean ± SD (*n* = 3).

Pathogenic bacteria can adhere to the surface of medical implants and form biofilms.^70^ When in contact with open wounds, the planktonic bacteria released from such biofilms cause severe infection and tissue damage, leading to impeditive wound healing.^71,72^ Thus, an ideal wound dressing should be able to prevent bacterial adhesion efficiently. Because the bottom surface of the as-prepared films was supposed to directly cover the wounds, the anti-adhesion activity of this layer was investigated using the colony counting method.^73^ As shown in Fig. 6, compared with the PVDF film, much lower numbers of bacteria adhered to the bottom surfaces of the PVDF/NS25 and PVDF/NS50 films after co-incubation with *A. baumannii*, which indicated that the NS incorporated into these films could effectively fight against bacteria and hinder their binding.^21^ In addition, the PVDF/NS25 film reduced the adherence of bacteria compared with the PVDF/NS50 film, which might be due to its increased surface hydrophobicity (Fig. 2B).^74,75^ Overall, the bottom surface of PVDF/NS25 showed visible superiority for anti-bacterial adhesion.

### 3.7 *In vitro* cytotoxicity of PVDF/NS films

**Fig. 7:**
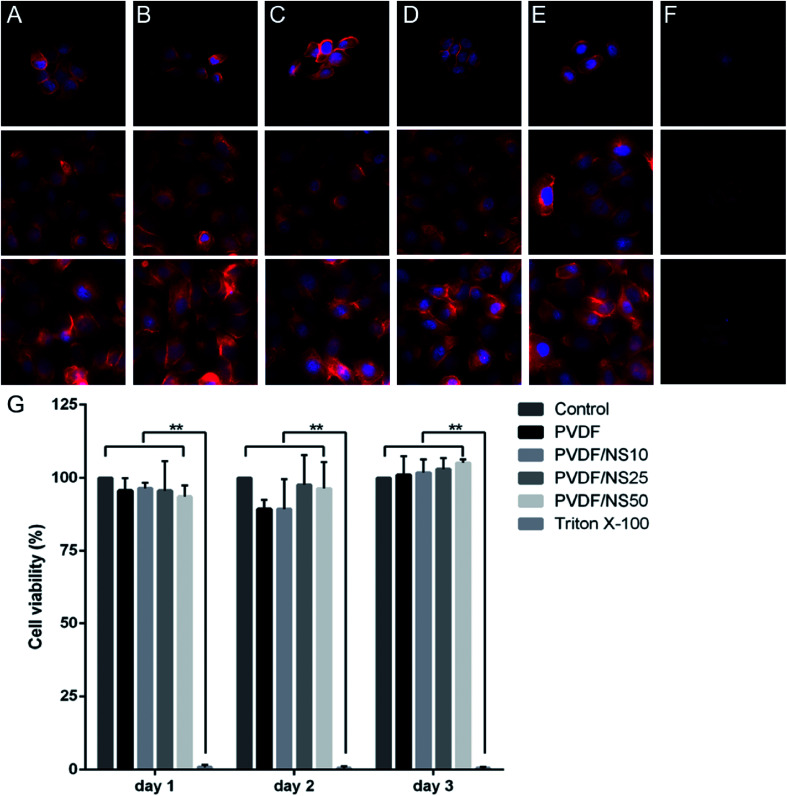
Cytotoxicity assessment of the PVDF, PVDF/NS10, PVDF/NS25 and PVDF/NS50 films. Fluorescence microscopy images of HaCaT cells treated with nothing (A), or leach liquor obtained from (B) PVDF, (C) PVDF/NS10, (D) PVDF/NS25, (E) PVDF/NS50 films, or (F) 0.5%(v/v) Triton X-100 after 1, 2, and 3 days. (G) Corresponding cell viability of HaCaT cells measured using the CCK8 test.

Biocompatibility is a crucial factor for a wound dressing applied to the human body, so the biotoxicity of the PVDF/NS films was evaluated toward HaCaT cells using cell morphology observations and a CCK8 assay. As shown in Fig. 7A–E, cells treated with the leach liquor obtained from PVDF, PVDF/NS10, PVDF/NS25 or PVDF/NS50 films showed similar morphologies to those in the negative control group. In addition, all the cells in these groups grew and spread well over time. However, the cells cultured in the positive control group (treated by 0.5% (v/v) Triton X-100, a surface active agent that damages the cell membrane) displayed disorganized shapes with a low density (Fig. 7F). Furthermore, the CCK8 result showed that no significant differences in cell viability were found among the PVDF, PVDF/NS10, PVDF/NS25, PVDF/NS50 and negative control groups at three determined time points, but the cell viability of the positive control group was obviously lower than that of the negative control group (Fig. 7G). These results indicated that pristine PVDF and functional PVDF/NS composites exhibited no obvious cytotoxicity to mammalian cells, which was in accordance with the data showing that the concentrations of Ag^+^ released from these films were far lower than the toxic concentration (Fig. 3).^62^

Based on the above results, the PVDF/NS25 film which possessed excellent antibacterial activity and antifouling activity as well as good biocompatibility was selected for the following *in vivo* animal experiment.

### 3.8 *In vivo* effect of the PVDF/NS25 film on wound healing

**Fig. 8:**
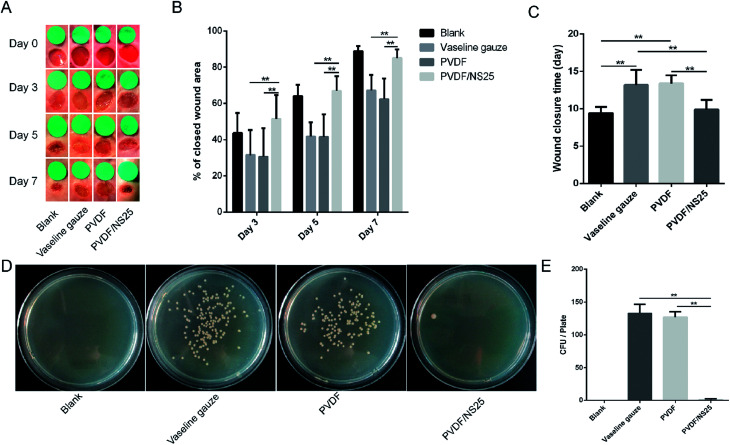
The effect of PVDF/NS25 on wound healing. (A) The representative macroscopic appearances of wounds from the Blank, Vaseline gauze, PVDF and PVDF/NS25 groups. (B) The areas of wound closure at days 3, 5 and 7 post-surgery. (C) The average wound closure times. (D) The photographs of bacterial CFU and (E) corresponding quantitative results. The values are shown as the mean ± SD (*n* = 5).

The effect of the PVDF/NS25 film on infectious wounds *in vivo* was investigated using a murine full-thickness skin defect wound model. Briefly, the Vaseline gauze, PVDF and PVDF/NS25 films pre-seeded with *A. baumannii* bacteria were each used to cover the wounds, then the healed wounds were photographed at determined time points and the corresponding closed wound areas were measured. The wound treated with nothing was set as the Blank group. As shown in Fig. 8A, plenty of purulent exudation was found in the wounds treated with Vaseline gauze or PVDF at day 3 post-surgery, and the skin around these wounds was red and swollen, indicating severe bacterial infection. However, the wounds treated with the PVDF/NS25 film remained as neat and tidy as the Blank group (without bacterial inoculation), and visible newly-generated epidermis migrated from the wound edge to the wound center. At days 5 and 7, obvious wound festering was still observed in the Vaseline gauze and PVDF groups, but the wound treated with the PVDF/NS25 film was almost healed. Correspondingly, the quantitative results of wound closure showed that the closed wound area in the PVDF/NS25 group was significantly larger than those in the Vaseline gauze and PVDF groups at all determined time points (Fig. 8B). After 7 days, the wound closure rates in the Vaseline gauze and PVDF groups were 67.1% and 62.4%, respectively, whereas the rate was up to 85% in the Blank and PVDF/NS25 groups. Furthermore, the average wound closure times for the Vaseline gauze- and PVDF film-treated mice were 13.2 days and 13.4 days, respectively, while it was only about 9.9 days for the PVDF/NS25-treated mice, which was nearly the same as the wound closure time for the mice in the Blank group (9.4 days) (Fig. 8C). To quantify the *in vivo* antibacterial efficacy of the PVDF/NS25 film, the wounds of mice at day 7 post-surgery were harvested and homogenized, and the bacterial number was counted by the plate counting method. As shown in Fig. 8D and E, the bacterial number in the PVDF/NS25 group was significantly lower than those in the Vaseline gauze and PVDF groups, which further demonstrated the *in vivo* antibacterial activity of the PVDF/NS25 film. These results indicated that PVDF/NS25 could effectively prevent bacterial invasion induced by the biomaterial implantation *in vivo*, eventually accelerating wound healing and shortening the healing time.

**Fig. 9:**
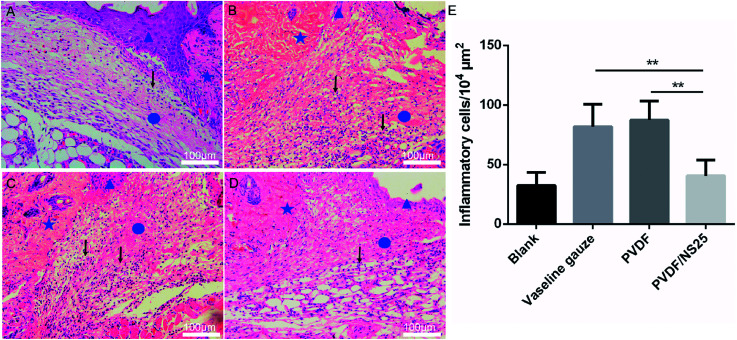
The inflammatory cells infiltrating the wound edge at day 3 post-surgery. Representative H&E staining images of the (A) Blank, (B) Vaseline gauze, (C) PVDF and (D) PVDF/NS25 groups. The pentagram indicates undamaged skin tissue, the circle indicates the wound area, and the triangle indicates the newly formed epidermis. The black arrows indicate the inflammatory cells. (E) Number of inflammatory cells infiltrating the subcutaneous areas of the wounds. The values are shown as the mean ± SD (*n* = 4). Scale bars: 100 μm.

To further evaluate the *in vivo* anti-infective activity of the PVDF/NS25 film, the wound tissue was harvested and stained by H&E. The histological images showed that abundant inflammatory cells infiltrated the subcutaneous areas of wounds treated by the Vaseline gauze and PVDF films, while such a sign of infection was barely observed in the same region of the wound treated by the PVDF/NS25 film (Fig. 9A–D). Moreover, the quantitative result revealed that the number of inflammatory cells in the wound edge of the PVDF/NS25 group was far lower than that in the Vaseline gauze or PVDF groups (Fig. 9E). The number of inflammatory cells in the PVDF/NS25 group was slightly higher than that in the Blank group (*P* > 0.05); this was probably because the wounds of the PVDF/NS25 group were treated with the bacteria-seeded samples, and the initial wound healing stage was inevitably disturbed by the bacteria as previously described.^14,76^ Accordingly, this phenomenon further confirmed that the PVDF/NS25 film efficiently protected the wound from bacterial infection.

**Fig. 10:**
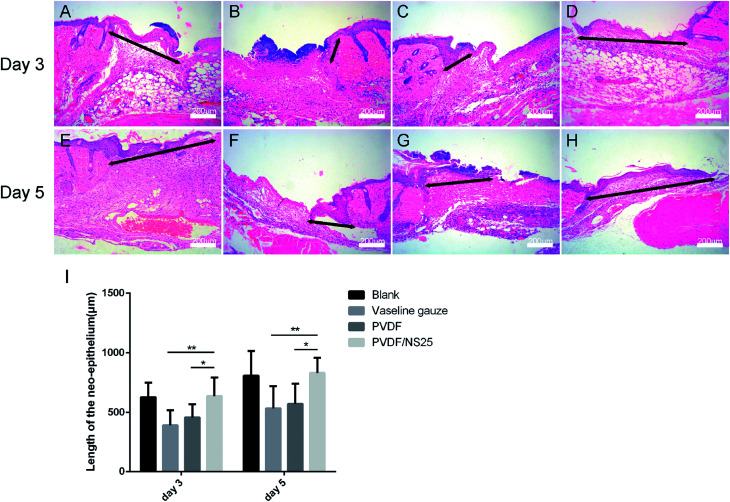
The effect of PVDF/NS25 on re-epithelialization. Representative histological images of the length of the newly generated epidermis at day 3 and day 5 post-surgery in the (A and E) Blank, (B and F) Vaseline gauze, (C and G) PVDF and (D and H) PVDF/NS25 groups. The black double-headed arrows indicate the length of the neo-epithelium. (I) Measurements of the length of the neo-epithelium at days 3 and 5 post-surgery. The values are shown as the mean ± SD (*n* = 4). Scale bars: 200 μm.

**Fig. 11:**
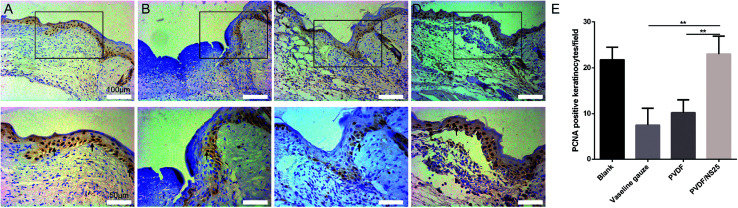
The PCNA-positive keratinocytes in wound tissue at day 3 post-surgery. Representative images of immunohistochemical staining of PCNA in the (A) Blank, (B) Vaseline gauze, (C) PVDF and (D) PVDF/NS25 groups. The rectangular insets represent magnified areas, and the black arrows indicate PCNA-positive keratinocytes. (E) The number of PCNA-positive keratinocytes per field in newly generated epidermis. The values are shown as the mean ± SD (*n* = 4).

As is well known, re-epithelialization is a necessary factor for wound healing. Fast re-epithelialization is beneficial for closing the wound surface at the early stage and reducing hypertrophic scar formation at the late stage of the wound healing process.^2,77^ Thus, the effect of PVDF/NS25 on re-epithelialization was further histologically analyzed. Fig. 10 shows that the length of newly-generated epidermis was significantly longer in the PVDF/NS25 group than that in the Vaseline gauze or PVDF groups both at day 3 and day 5 post-surgery, which was in line with the macroscopic wound appearance (Fig. 8A). No significant difference was observed in the length of the newly regenerated epidermis between the PVDF/NS25 group and the Blank group. This indicated that the PVDF/NS25 film could promote re-epithelialization more effectively than Vaseline gauze or PVDF film when used to cover the wounds; the effective action was probably due to the excellent antibacterial and antifouling activities as well as the suitable WVTR. Furthermore, because keratinocyte proliferation plays an important role in the re-epithelialization process, PCNA protein (cell proliferation marker) was then assessed using immunohistochemistry. Consistently, many more PCNA positive keratinocytes were found at the wound edge in the PVDF/NS25 group than in the Vaseline gauze or PVDF groups (Fig. 11), which demonstrated that PVDF/NS25 could promote keratinocyte proliferation to accelerate re-epithelialization *in vivo*. Taken together, all these results indicated that the PVDF/NS25 film could prevent the *in vivo* bacterial infection caused by biomaterial contamination and maintain a moist microenvironment to facilitate the wound healing process.

### 3.9 *In vivo* biocompatibility evaluation

The potential *in vivo* toxicity of NS-based materials is a great concern. To evaluate the *in vivo* biocompatibility of our PVDF/NS25 film, a histological analysis of the major organs of mice was carried out.^45,46^ As shown in Fig. S3 (ESI†), compared with the control and clinical Vaseline gauze groups, no appreciable abnormalities or destruction of the heart, liver, spleen, lung and kidney were observed after 7 days for the PVDF/NS25 group, indicating that our optimal film had negligible biotoxicity towards normal tissues. This result further demonstrated that PVDF/NS25 has a great potential for application in clinical practice.

## 4. Conclusions

In the present study, we report an ideal wound dressing prepared by integrating NS into a porous PVDF film using an immersion phase inversion method. The composite showed satisfactory antibacterial activity against *A. baumannii* and *E. coli* without any observed toxicity to mammalian cells. More importantly, the nanoporous top layer of this composite exhibited stable anti-permeation activity toward bacteria, while the microporous bottom layer could efficiently prevent bacterial infection caused by surface biofouling when the wound was covered. These results indicate that PVDF/NS25 has a promising application in wound management.

## Conflicts of interest

The authors declare no competing financial interests.

## Supplementary Material

RA-008-C8RA03234C-s001
