# Correction: Janus *N*,*N*-dimethylformamide as a solvent for a gradient porous wound dressing of poly(vinylidene fluoride) and as a reducer for *in situ* nano-silver production: anti-permeation, antibacterial and antifouling activities against multi-drug-resistant bacteria both *in vitro* and *in vivo*

**DOI:** 10.1039/d4ra90102a

**Published:** 2024-10-02

**Authors:** Menglong Liu, Ying Wang, Xiaodong Hu, Weifeng He, Yali Gong, Xiaohong Hu, Meixi Liu, Gaoxing Luo, Malcolm Xing, Jun Wu

**Affiliations:** a Institute of Burn Research, State Key Laboratory of Trauma, Burn and Combined Injury, Southwest Hospital, Third Military Medical University (Army Medical University) Chongqing 400038 China logxw@yahoo.com malcolm.xing@umanitoba.ca editorinchief@burninchina.com +86-23-65461677 +86-23-68754173; b State Key Laboratory of Polymer Materials Engineering, Polymer Research Institute of Sichuan University Chengdu 610065 China; c Department of Burns, The First Affiliated Hospital, SunYat-Sen University Guangzhou 510080 China; d Department of Mechanical Engineering, University of Manitoba Winnipeg MB R3T 2N2 Canada

## Abstract

Correction for ‘Janus *N*,*N*-dimethylformamide as a solvent for a gradient porous wound dressing of poly(vinylidene fluoride) and as a reducer for *in situ* nano-silver production: anti-permeation, antibacterial and antifouling activities against multi-drug-resistant bacteria both *in vitro* and *in vivo*’ by Menglong Liu *et al.*, *RSC Adv.*, 2018, **8**, 26626–26639, https://doi.org/10.1039/C8RA03234C.

The authors regret that an incorrect version of Fig. 1 was included in the original article. The correct version of Fig. 1 is presented below.

**Fig. 1:**
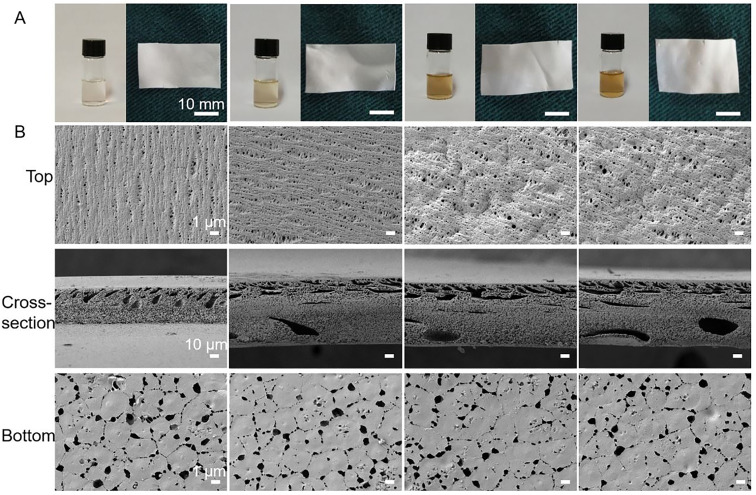
(A) Macroscopic appearances of solutions and the corresponding formed films of PVDF, PVDF/NS10, PVDF/NS25 and PVDF/NS50 (from left to right) after 24 h incubation. (B) SEM images of PVDF, PVDF/NS10, PVDF/NS25 and PVDF/NS50 films. Magnification of top surface and bottom surface images: ×3000; magnification of cross-section images: ×300.

The Royal Society of Chemistry apologises for these errors and any consequent inconvenience to authors and readers.

