# Supplementary material for: Janus N,N-dimethylformamide as a solvent for a gradient porous wound dressing of poly(vinylidene fluoride) and as a reducer for in situ nano-silver production: anti-permeation, antibacterial and antifouling activities against multi-drug-resistant bacteria both in vitro and in vivo
Source: RSC Adv. 2018 Jul 25;8(47):26626–39. doi: 10.1039/c8ra03234c (PMC9083098; doi:10.1039/c8ra03234c)
Supplement: RA-008-C8RA03234C-s001 [file RA-008-C8RA03234C-s001.pdf]

Electronic Supplementary Material (ESI) for RSC Advances.
This journal is © The Royal Society of Chemistry 2018

Supplementary material

Janus *N,N*-Dimethylformamide as solvent for gradient porous wound dressing of poly(vinylidene fluoride) and as reducer for *in situ* nano-silver production: anti-permeation, antibacterial and antifouling activity against multi-drug-resistant bacteria from *in vitro* to *in vivo*

Menglong Liu[1], Ying Wang[1], Xiaodong Hu[2], Weifeng He[1], Yali Gong[1], Xiaohong Hu[1], Meixi Liu[1], Gaoxing Luo[1*], Malcolm Xing[1,4*], Jun Wu[1,3*]

[1] Institute of Burn Research, State Key Laboratory of Trauma, Burn and Combined Injury, Southwest Hospital, Third Military Medical University (Army Medical University), Chongqing 400038, China

[2] State Key Laboratory of Polymer Materials Engineering, Polymer Research Institute of Sichuan University, Chengdu, 610065, China.

[3] Department of Burns, the First Affiliated Hospital, SunYat-Sen University, Guangzhou 510080, China.

[4] Department of Mechanical Engineering, University of Manitoba, Winnipeg MB, R3T 2N2, Canada.

* Corresponding authors:

Gaoxing Luo, E-mail address: logxw@yahoo.com;

Malcolm Xing, E-mail address: malcolm.xing@umanitoba.ca;

Jun Wu, E-mail address: editorinchief@burninchina.com, Tel: 0086-23-68754173, Fax: 0086-23-65461677.

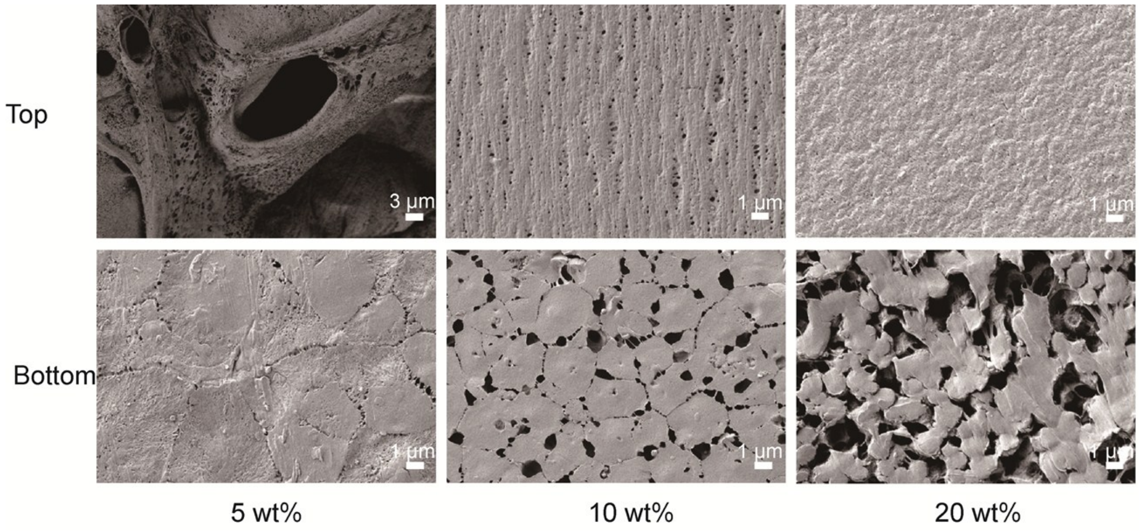

Fig. S1. Morphology of PVDF films formed with different polymeric concentrations using phase inversion method.

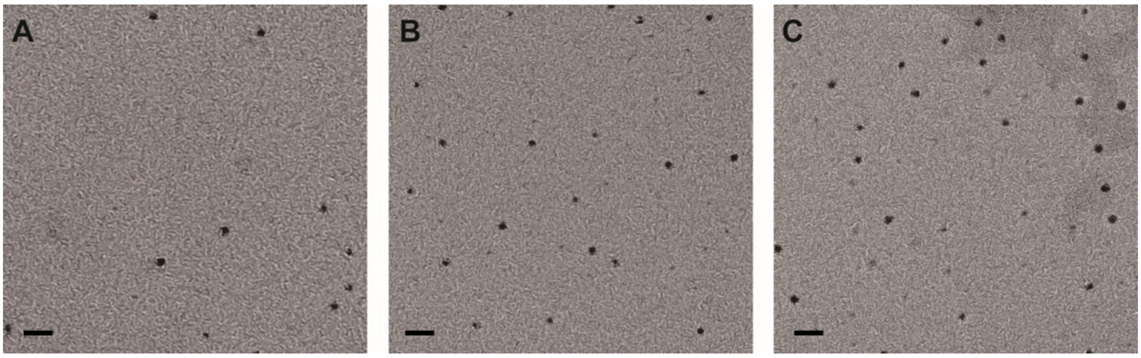

Fig. S2. The TEM images of NS from (A) PVDF/NS10, (B) PVDF/NS25 and (C) PVDF/NS50 solutions after 24 h incubation. Scale bars: 20 nm.

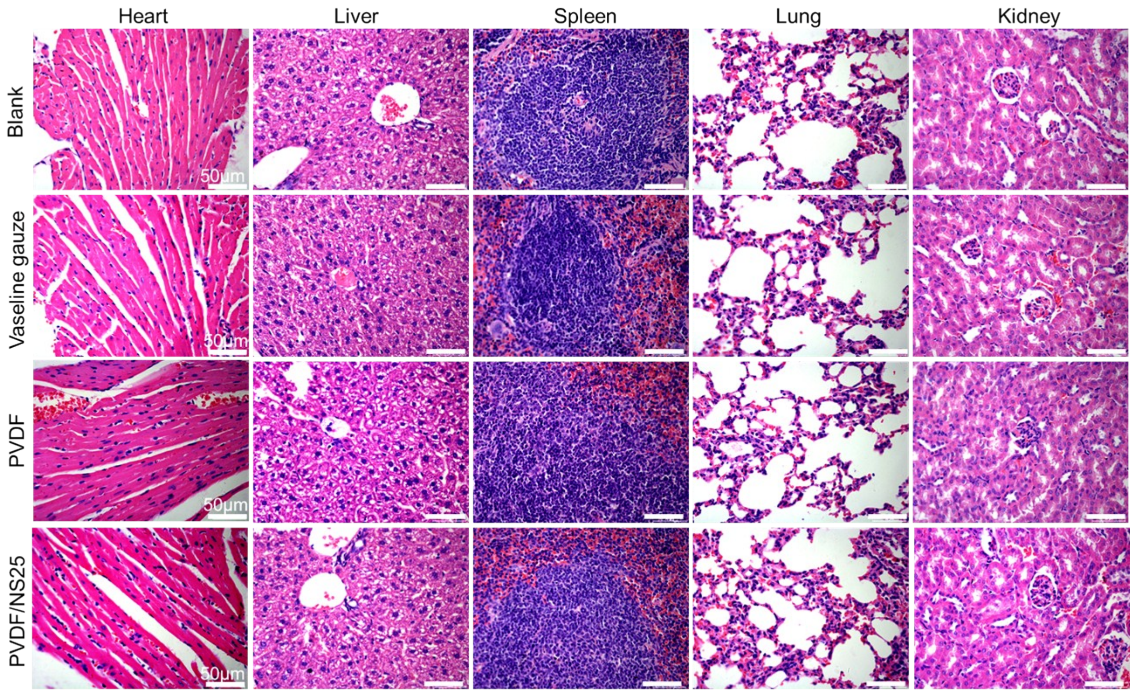

Fig. S3. The histological images of heart, liver, spleen, lung and kidney obtained from mice treated by nothing, Vaseline gauze, PVDF and PVDF/NS25 films after 7 days.
